# Evaluation of a specific system of extended producer responsibility for veterinary medicines packaging waste

**DOI:** 10.1177/0734242X251326270

**Published:** 2025-03-27

**Authors:** Graça Martinho, Ana Gomes, Pedro Santos, Mário Ramos

**Affiliations:** 1 MARE – Marine and Environmental Sciences Centre, Associate Laboratory ARNET – Aquatic Research Network, Portugal; 2 Department of Environmental Sciences and Engineering, NOVA School of Science and Technology, NOVA University Lisbon, Caparica, Portugal

**Keywords:** Veterinary medicine (VM), packaging waste, extended producer responsibility (EPR), VM waste composition, potential for reduction, potential for recycling

## Abstract

In Portugal, packaging products for veterinary medicines (VM) are subjected to the extended producer responsibility (EPR) scheme coordinated by a Producer Responsibility Organisation (PRO), responsible for the management of both human and VM packaging waste. Despite an 80% recycling target for VM packaging waste, recent years have consistently shown performance below this level. However, there is no compositional data on VM packaging waste in scientific literature, hindering effective problem diagnosis and solution proposals. So, this research proposes a protocol to characterise VM packaging waste entering and leaving a sorting centre and presents the corresponding results. Of the 822.1 kg entering the centre, glass is the predominant material (66.7%, in weight). Often, glass has rubber and metal attached, but this is not recognised as a constraint on recyclability by the glass recycling industry. Biohazardous VM waste was found in the containers dedicated to pharmacologic VM waste, raising a challenge. To evaluate alignment with the principles of circularity, opportunities for waste reduction were assessed but found to be limited by stringent VM regulations. Nevertheless, the potential for recycling could be enhanced through adjustments to the sorting procedures. Moreover, future research should prioritise biohazard risks and operational aspects of recyclability. In addition, discussion and potential reconsideration of recycling rate targets for this waste category are recommended.

## Introduction

### Veterinary medicines and packaging waste

Despite well-known and widely described environmental impacts, the growing use of veterinary medicines (VM) is justified, above all, to protect and or improve animal health (Nightingale et al., 2023, Tasho and Cho, 2016). However, it is necessary to consider the practices adopted in a real-world context, namely those related to the knowledge acquired through habits, with relevance to social-cultural factors and decision-making processes based on experimental practices overtime (Rees et al., 2021).

To better understand the results obtained in studies involving animals and the consequences to human life, such as the transference of VM to ecosystems, researchers have observed these outcomes over an extended period (Diepens et al., 2023; Kuppusamy et al., 2018; Li et al., 2017; Wang et al., 2016). These studies consider various aspects, including soil contamination (Albero et al., 2018; Cooper et al, 2015; Pinheiro et al., 2013), effects on plants (Tasho and Cho, 2016), wastewater impacts (Zhi et al., 2020), the discharge of effluents from livestock farming into catchments (Hanamoto et al., 2023) and the influence on waste treatment (Gaballah et al., 2024). Additionally, assessment took place throughout the COVID-19 pandemic (Decaro et al., 2020).

As identified by Koytcheva et al. (2021), VM must include proactive methods and communication focused on environmentally friendly practices. However, it is relevant to mention the absence of scientific studies in the veterinary field pursuing of this goal, even though scientific advances exist. For example, experiments have proven that inexpensive solutions can enable the removal of VM from aqueous solutions, as demonstrated by Nguyen et al. (2020). Furthermore, toxicology research is exploring new approaches and methods, substituting animals with cell cultures, as stated by Antonine et al. (2022).

Studies regarding VM waste management are scarce, and the existing regulations in the industry do not resolve recently identified environmental challenges. In Pakistan, risk assessments and practices to reduce the impact of and better manage biological and VM waste are recorded as not being considered (Qasmi and Khan, 2019). Although less recently, this was also the case in South Africa where, between 2001 and 2003, incineration was the most common waste treatment option, despite the availability at the time of other technologies with better environmental impacts (McLean et al., 2007). Moreover, in both cases, the appropriate separation of VM packaging waste at the source was missing, as was the existence of proper documentation for the management of this type of waste (Qasmi and Khan, 2019; McLean et al., 2007).

Considering veterinary practices, most of the personal protective equipment used when managing drugs or veterinary hazardous waste, is disposed of as mixed waste (Huertas et al., 2019), instead of fulfilling other hierarchically superior waste management options. There is also a recognised need to introduce or reinforce legal requirements for medicinal waste, including veterinary waste (Qasmi and Khan, 2019; McLean et al., 2007). However, sustainability-related topics are still lacking professional veterinary input because there are no dedicated elements in the VM curriculum raising awareness of the topic, for instance, the impact of veterinarian practices on climate change (Kramer et al., 2020).

In addition, an intensification of the circular economy principles along the pharmaceutical packaging value chain is necessary, including the management of VM packaging waste, where the security of medicines is prioritised, controlled through a strict specific legal framework (Schmerold et al., 2023). Notably, robust scientific literature on this subject is practically absent, and more research initiatives are needed because packaging problems in the field are increasing, and new visions and strategies are necessary to accomplish circularity (Lima et al., 2023; Somlai et al., 2023; European Commission, 2020). Furthermore, it is necessary to resolve scientific knowledge gaps not only to encounter operational solutions to the waste management of packaging composed of different materials but also to encourage cooperation between stakeholders to resolve these challenges (Salmenperä et al., 2022).

### European legal framework for packaging waste and practices in some European Member States

The packaging for VM is a complex subject because it is composed of primary, secondary and tertiary packaging layers, with the main function being to protect and safeguard medicines. But it is also a difficult theme because packaging can be composed of multiple distinct materials, such as glass, different plastic polymers, metals, rubber, cardboard, paper or composite materials (Dolci et al., 2025; Salmenperä et al., 2022). Moreover, it is challenging to change packaging within this system because there are strict regulations and marketing permits to comply with (Salmenperä et al., 2022).

To better manage the packaging waste stream, the European Union introduced the Directive on Packaging and Packaging Waste (Directive 94/62/EC, of 20 December), establishing that countries from the European Union should guarantee that producer responsibility schemes are established for all packaging by the end of 2024, but the performance outcomes may differ from country to country, potentially leading to poor comparability (Somlai et al., 2023).

Specific targets were established for the recycling of different materials, for three distinct periods: the present, 2025, and 2030. The targets are respective to the aforementioned periods: all-packaging (55%, 65% and 70%), glass (60%, 70% and 75%), plastic (25%, 50% and 55%) and paper and cardboard (60%, 75% and 85%).

The European Union Strategic Approach to Pharmaceuticals in the Environment (COM(2019) 128 final, of 11 March 2019) establishes the importance of improving the management of waste, including veterinary waste. For instance, this strategy refers to the need to exchange best practices to promote the proper collection and disposal of this type of waste, namely through extended producer responsibility (EPR). Additionally, Regulation (EU) 2019/6, of 11 December 2018 on veterinary medicinal products establishes that the European Member States shall provide adequate systems to collect and dispose of the waste resulting from veterinary products, in this case aiming to protect ecosystems and human and animal health.

Moreover, according to the European Platform for the Responsible Use of Medicines in Animals (EPRUMA, 2021), a European organisation comprising a group of diverse European stakeholders (e.g. veterinarians, farmers, manufacturers of animal medicines, professionals working in animal health, among other), which aims to prevent and control animal diseases through the proper use of VM, there are different practices in Europe regarding waste disposal of VM, although none of them following the strategy mentioned above.

For instance, in Estonia, VM are delivered to pharmacies, to be collected in special containers, stating ‘extra dangerous items’; in Finland, the obligation for proper waste management relies on the veterinarian, taking into account that medicines are hazardous waste; in Germany, the municipal waste disposal scheme can be used in most local communities since the destination for this waste is incineration; in Ireland, the veterinary waste is sent to incineration via the proper waste stream; in Slovenia, there is a specific regulation for the disposal of VM; in Sweden, leftover VM are sent for disposal or to pharmacies, who are also responsible for sending them for this type of treatment and in the United Kingdom, this waste is separated into hazardous and non-hazardous streams, although incineration is the destination in both cases, they comply with different operational requirements.

Portugal has an EPR system specifically dedicated to the packaging and packaging waste of VM products, managed by a specific producer responsibility organisation (PRO) named Valormed (2022). Despite high-quality glass being received in good condition by the sorting centre (Valormed, 2022), national regulations make the recycling process difficult to implement relative to other waste streams.

### Background and main objectives

In Portugal, even with a specific EPR scheme for VM waste, the recycling rate for VM packaging waste remains low compared to the mandatory targets established by the national waste authority. This is enforced through a specific license issued through the Portuguese PRO (Valormed, 2022). However, this problem is, in part, a result of the involvement of different entities in the decision-making process, each with distinct liabilities. The representatives of the glass industry consulted during this research declare no operational problems when recycling the glass from this waste stream, even when it includes a mixture of glass with metal and rubber, since most veterinary packaging has combinations of these materials.

To better understand the problem and substantiate operational and political decisions, in terms of improving the indicators related to the recycling of VM packaging waste, it is essential to begin by assessing the waste that is collected. But currently there is no compositional data about this waste stream. In Portugal, it is only mandatory to periodically obtain compositional data for municipal waste, for which there is a specific regulation that determines the rules for its physical characterisation (e.g. waste sample, waste categories, periodicity). However, there is no such regulation for VM packaging waste. This is relevant because waste characterisation provides information both to better address a problem or issue, and to decide on the best alternatives for its management (Lagerkvist et al., 2010).

So, this research aimed to obtain a mass balance, comparing the data and the physical characterisation of the VM packaging waste collected which enters the respective sorting centre, and the waste which leaves the facility after this sorting process (refuse). Acquiring knowledge about the physical composition of this waste stream is innovative, this being the first time it was attempted in Portugal, and with scientific data on this specific topic not being found in the scientific literature.

These results also facilitate discussions on waste reduction and the recycling potential of VM packaging waste, providing valuable insights for both operational and political decision-making. Specifically, this is crucial when tackling circular principles concerning this type of waste, relying on comparable indicators to deliver a robust assessment, especially within the European context.

## Portuguese extended producer responsibility for veterinary medicines packaging waste

VM packaging waste is part of the specific flow of packaging waste that uses a management model based on the principle of EPR. Following the European Directive on Packaging and Packaging Waste, and considering the current knowledge of the authors, Portugal is the only European country that opted to create an integrated system dedicated exclusively to the management of packaging for human and VM, given the specificity of this waste and its risks to human health. In 1999, the pharmaceutical industry created a PRO, named Valormed – *Sociedade Gestora de Resíduos de Embalagens e Medicamentos, Lda.*, a non-profit society. This PRO is responsible for an Integrated System for Waste Management of Packaging and Medicines (*Sistema Integrado de Gestão de Resíduos de Embalagens e Medicamentos*-SIGREM), comprising two subsystems, one for human medicines, implemented in 2000, and one for VM, implemented in 2008 (Valormed, 2022).

**Figure 1.**
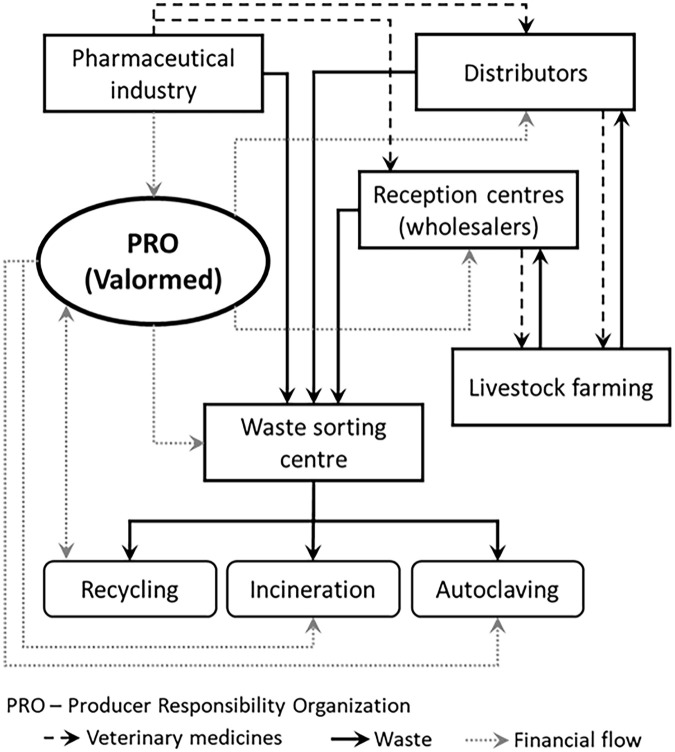
Integrated system for veterinary medicines packaging waste, in Portugal. Source: adapted from Valormed (2022).

The Portuguese Environment Agency (*Agência Portuguesa do Ambiente*), as the national waste authority, regulates SIGREM, and through licensing establishes collection, recycling and valorisation targets (Dias-Ferreira et al., 2016) for these two subsystems. Alongside that, the Portuguese National Authority for Animal Health, the Directorate General for Food and Veterinary (DGAV) (*Direção Geral de Alimentação e Veterinária*), is the national authority issuing VM commercialisation licences. A schematic representation of the SIGREM, specifically for the VM subsystem, is presented in Figure 1.

In summary, SIGREM has three main flows (Valormed, 2022): VM products, VM packaging waste and financial flows. Concerning the flow of VM: wholesalers purchase VM from the pharmaceutical industry directly or through distributors, and then they are sold to the livestock farming industry. After VM is administered to animals, packaging waste is segregated at livestock farms and returned to reception centres/distributors across Portugal; VM packaging waste stored in reception centres is collected and sent to a sorting centre where it is processed and sent for recycling, incineration or physico-chemical treatment operators; packaging waste generated by the pharmaceutical industry is sent directly to sorting centres. Regarding financial flows: like other EPR systems, the fee-paying entities are those that put packaging products into the market (Hahladakis et al., 2018; Pires et al., 2015), namely the pharmaceutical industry. In this case, it is possible for Valormed to fund VM packaging waste management, including waste collection and treatment. Fees paid by the pharmaceutical industry are also used by Valormed to fund communication campaigns and research projects.

Regarding VM packaging waste collection, Valormed provides reception centres with two different containers: biological and pharmacological. Biological containers are smaller and are specifically designed to contain only immunological VM packaging waste (i.e. vaccines packaging), which is considered biohazardous waste by Portuguese law. There is mandatory segregated collection for its physical-chemical pre-treatment (e.g. autoclaving, microwave treatments), followed by disposal in non-hazardous landfill, or through incineration. Other VM packaging waste is put in pharmacological containers. It is mandatory to segregate the packaging waste at livestock farms or reception centres.

Currently, the VM packaging waste is transported to the sorting centre, where both human medicines and VM are separated. Given that the collection frequency and quantity of VM is lower than that for human medicines, the sorting process does not justify continuous processing. Therefore, the procedure is to store VM packaging waste until there is a sufficient quantity to justify the sorting line functioning for at least 1 day of operation.

In practice, sorting VM packaging waste for recycling is only carried out for glass with volumetry equal to or greater than 100 ml, the plastic bags used to package containers, and the paper/cardboard of the containers themselves. The remaining VM (glass with volumetry of less than 100 ml, plastic packaging, paper/cardboard, waste outside the VM subsystem and other waste) is sent for incineration.

Regarding the operational component of the sorting centre, VM packaging waste collected by Valormed has increased since its implementation. In 2022, around 76.2 tonnes were collected at the reception centres (Valormed, 2022). The license conceded to Valormed established a target to recycle 80% of the collected packaging. The data presented in the 2018 report indicates an overall recycling rate of 55.3%. The recycling rate for the human medicines subsystem was 58.6%, whereas the VM subsystem did not exceed 21.9% (Valormed, 2022). The amount of VM waste managed through other ways, outside the Valormed subsystem, such as municipal waste collection and disposal (e.g. drop-off systems) is unknown. The deviation from the defined target served as the primary motivation for the current research, aimed at understanding challenges and determining appropriate actions.

## Methods

### Conceptualisation and criteria for the characterisation of veterinary packaging waste

For this VM packaging waste assessment, in Portugal, two waste characterisation campaigns took place: the main objective of the first was to assess the characteristics of a sample of VM packaging waste entering the sorting centre, also establishing the representativeness of the sample for future field surveys in Portugal or elsewhere; the second, concerned the VM packaging waste refuse leaving the sorting centre, in this case, to better understand the problem and the challenges regarding the treatment of this type of waste, allowing the study of the refuse at the sorting centre and the creation of a mass balance.

For the campaigns, stratified random sampling was used, as waste is regarded as inhomogeneous (Lagerkvist et al., 2010). The Portuguese NUTS II regions were considered as the number of strata, and in each stratum Valormed reception centres for VM packaging waste were identified, where VM waste is collected from producers. The contribution of each stratum was based on waste collected by weight over 3 years, between 2013 and 2015, specifically: North (36.1%), Alentejo (25.0%), Centre (27.8%), Lisbon Metropolitan Area (LMA) (8.3%) and Autonomous Region of Azores (2.8%). Regarding the Portuguese territory, there were no reception centres from the regions of the Algarve and the Autonomous Region of Madeira.

### Veterinary packaging waste entering the sorting centre

#### Veterinary medicines packaging waste characterisation

To characterise the waste entering the sorting centre, a sample of 80 containers was considered, with 76 pharmacological containers and 4 biological containers.

For quantifying and characterising VM packaging waste entering the sorting centre, integrating the EPR subsystem, the field survey aimed to assess and catalogue the following criteria: type of material (i.e. plastic – specifying the polymer, glass, metal, paper/cardboard or composites); dimension categories (i.e. volume, weight or other) and commercial name, to classify VM packaging waste into medicine groups. Additionally, if packaging contained medicines, remnants were collected. Other packaging waste, either that beyond the Valormed VM subsystem, or other waste that did not fit the aforementioned categories was identified and registered (e.g. needle/syringe, veterinary gloves, UV lamps).

For VM packaging waste from the Valormed subsystem, the name of the medicine, as indicated on packaging labels, was registered. Moreover, the VM packaging waste was categorised according to the Anatomical Therapeutic and Chemical groups (ATCVet) using commercial names and the Medvet (2016) database, provided by DGAV.

#### Sample representativeness

Since this was the first physical characterisation conducted, there was no established protocol for classifying this type of waste. Therefore, it was not possible to assess the variability in packaging waste for VM. In this context, and considering other waste stream characterisation campaigns, to determine an adequate sample size, the study assumed: a 10% level of precision and confidence levels of 95% (Newbold, 1995) and 90% (Chancerel and Rotter, 2009). It also considered the following assumptions: sampling units corresponded to each container (either pharmacological or biological), analysis was made by material type and data were processed as the percentage of the weight of each container. The sample size was calculated by using equation (1) (Chancerel and Rotter, 2009):

(1) $$N={\left(\frac{Z_{\left(1-\frac{\propto }{2}\right)}*S}{\Delta }\right)}^{2}$$

where N is the sample size; $Z_{\left(1-\frac{\propto }{2}\right)}$ is the desired level of confidence (tabulated); S is the standard deviation and Δ is the desired level of precision.

All data were treated with *Microsoft Excel* and *IBM SPSS Statistics*. The statistical results presented in Table 1 were obtained by considering the percentage distributions of the type of material (measured in weight), within each of the 80 sampled containers. It was concluded that the sample is representative for glass, for the two confidence intervals, because a superior number of containers were characterised in the two campaigns executed.

**Table 1.** Sample statistical data (Valormed and non-Valormed waste).

Waste material	Statistical data (%)							
	Minimum	Maximum	Mean	Median	SD	Precision	N (95%)	N (90%)
Glass	2.1	99.4	60.0	62.1	26.9	6.0	77	54
Plastic	0.3	87.6	26.6	22.6	20.1	2.7	218	154
Paper/cardboard	0.0	29.2	5.7	3.1	7.1	0.6	596	420
Composite	0.0	35.8	6.4	4.3	7.7	0.6	555	391
Metal	0.0	39.3	4.5	2.1	8.0	0.5	1,214	855
Refused waste	0.0	60.6	3.9	1.6	9.5	0.4	2,289	1,612
Other non-packaging waste	0.0	7.2	1.4	0.6	1.8	0.1	663	467
VM remnants	0.2	17.3	2.6	1.1	3.6	0.3	738	520
Other	0.1	9.7	3.6	2.2	4.3	0.4	552	389

SD: standard deviation; *N*: sample size (number of containers); VM: veterinary medicines.

### Veterinary packaging waste refuse

The criteria used to characterise the VM packaging waste involved collecting 80 containers and transporting them to the sorting centre, where the VM packaging waste was manually separated. The process of separating the VM waste followed the normal operating conditions for this type of waste at the sorting centre: manually separating the glass packaging with dimensions equal to or greater than 100 ml, the packaging bags and the subsystem paper/cardboard containers. After this separation was undertaken at the entrance of the sorting line, the remaining waste went via the conveyor belt to the sorting table, where waste was separated according to the sorting catalogue using the following criteria: the type of material (i.e. paper/cardboard, glass, plastic – separated by polymers, metal), the dimension categories and the condition of the packaging (whether it contained medication remnants or not).

### Mass balance

Through the information collected in the characterisation described in subsection ‘Veterinary packaging waste refuse’, and the operation described in section ‘Portuguese extended producer responsibility for VM packaging waste’, a mass balance was developed to complement and improve the assessment of this type of waste circuit in the sorting centre. Before the creation of the mass balance, it was necessary to gather data from the PRO, such as: operations involved in the REMV management subsystem, from the source, where the VM packaging waste is generated (producers), to their treatment (e.g. recycling, autoclaving, incineration); the quantities of VM packages placed on the market, taken up, sorted and sent for treatment; and the functioning and characteristics of the sorting centre.

The mass balance of the VM subsystem was carried out using data obtained in the characterisation campaign (subsection ‘Composition of the veterinary medicines packaging waste at the entrance of the sorting centre’), namely the component that was separated onsite, and that which went directly to refuse. The latter was separated by the authors for the purposes of this research. The mass balance was created considering an ideal situation of no losses within the separation circuit.

## Results and discussion

### Mass balance

**Figure 2.**
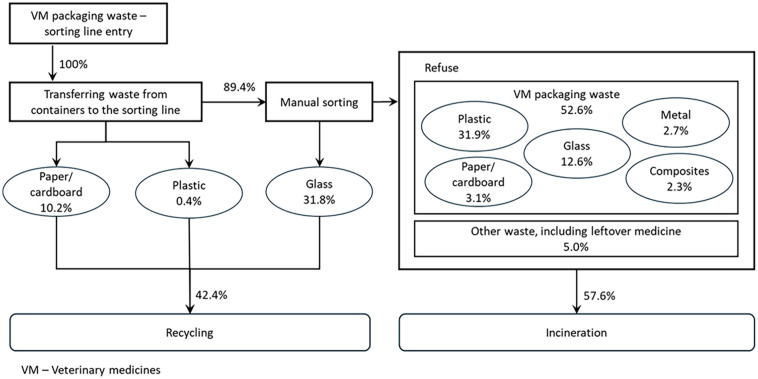
Mass balance at the sorting centre. SIGREM: System for Waste Management of Packaging and Medicines (*Sistema Integrado de Gestão de Resíduos de Embalagens e Medicamentos).*

To better understand the problems relevant to the management of this type of waste by the respective PRO, the initial results focus on the mass balance of the sorting centre (Figure 2). This objective was constructed by using information about the way the veterinary subsystem functioned and the results of the waste characterisation campaign, presented in subsection ‘ Composition of the veterinary medicines packaging waste at the entrance of the sorting centre’.

As observed, the current management entity theoretically sends 42.4% of the total VM packaging waste received for recycling, with the largest portion being glass packaging. Moreover, 31.8% of the total waste received is glass bottles with a capacity of 100 ml or higher, explaining the pre-existing separation of glass with this volumetry. These results must be analysed with caution, as they also include the bags (plastic) and cardboard boxes that make up the VM collection containers. However, when considering only the glass fraction, these results are higher compared to findings in the literature, where most of this type of waste was reported to be sent for incineration (Qasmi and Khan, 2019; McLean et al., 2007).

Concerning the refuse, the majority was plastic packaging, followed by glass smaller than 100 ml. The fraction of plastic packaging is more difficult to separate due to the different types of plastic found, as shown in subchapter subsection ‘Packaging size’. However, if the separation of high-density polyethylene (HDPE) packages equal to or greater than 100 ml was implemented (as in the case with glass), the percentage of the plastic component for recycling could be increased by 10% of the total.

### Composition of the veterinary medicines packaging waste at the entrance of the sorting centre

#### General characterisation

In general, the sample of 80 containers corresponds to 854.0 kg of waste entering the sorting centre. On average, each container weighed 10.7 kg. The minimum value registered was 1.4 kg (where plastic was the predominant material in the container), and 32.6 kg was the maximum value (with glass as the predominant material).

Contaminants within containers (e.g. waste from other packaging systems) represented 3.7% of the total weight (corresponding to 31.9 kg). The Central region of Portugal was the location where containers showed the most contaminant content (9.0%, measured in weight), and LMA, a more predominantly urban area, was the region that registered the least contaminant content (0.1%, measured in weight). Refuse waste stood out as the main category of total contaminants, representing 71.7%. Regarding non-veterinary packaging waste, plastic is the material that stands out (14.9%), followed by metal, glass and composites (12.1%, in total). Paper/cardboard material (1.2%) was considered in a separate category as it included packaging paper/cardboard and non-packaging paper/cardboard.

The results that follow concern the VM packaging covered by the Valormed subsystem (822.1 kg), excluding waste that is not covered (e.g. packages of other products or other types of waste).

#### Material composition

The results illustrating VM packaging waste composition, for the Valormed subsystem, are presented in Table 2. Glass is the predominant material in VM packaging waste (66.7%, in weight, and 47.5%, in number), being the sample representative for this material (subsection ‘Veterinary medicines packaging waste’), followed by plastic (25.8%, in weight, and 45.6%, in number). Composite materials, cardboard and paper, metal and other follows, although with less representativeness (in the subtotal, 6.4%, in weight and 7.0%, in number). Regarding cardboard and paper (2.5%, in number), it is a very low result relative to the expected values for this material, it being frequently used to group or wrap packaging that is directly in contact with VM. Furthermore, drug information leaflets are generally paper, which is part of almost all VM packaging waste. Therefore, it is assumed that this material will have been collected by other systems, for instance in the municipal waste segregated or unsegregated collection systems.

**Table 2.** Veterinary medicines packaging waste composition in the Valormed subsystem.

Categories of waste		Quantity			
		kg	%, in weight	Number	%, in number
**Packaging**	Glass	547.8	66.7	10,756	47.5
	Plastic	212.2	25.8	10,334	45.6
	Composite	19.2	2.3	779	3.4
	Cardboard/paper	17.4	2.1	564	2.5
	Metal	11.4	1.4	216	1.0
	Other	5.1	0.6	12	<0.1
VM remnants		9.0	1.1	-	-
Total		822.1	100.0	22,661	100.0

Few VM packaging units showed remnants (almost all were empty), in contrast with human medicines packaging waste studied in Portugal (Martinho and Santos, 2011), representing only 1.1% of the weight. This content predominantly related to expired VM, according to the information contained on the packaging.

#### Packaging size

These results are focused on packaging sizes for glass and plastic materials, as they are the most predominant materials in VM waste packaging, in the Valormed subsystem. Packaging size data was collected by registering the size (i.e. volume, dose or weight), if legible on the labels of each packaging.

Initially, glass distribution by packaging size is analysed (Table 3), with volume being the parameter that stands out (88.6% of all glass was defined by its volume). It was not possible to register size for only 2.6% of glass VM packaging waste due to missing labels or illegible units on labels.

**Table 3.** Glass distribution, according to the packaging size, in the Valormed subsystem.

Glass		Quantity (%)		
Volume (ml)	100	46.0	88.6
	250	14.4	
	50	10.3	
	500	3.8	
	200	3.1	
	Other	10.8	
	No data	0.2	
Dose		6.4	11.4
Weight (g)		2.4	
No data		2.6	
Total		100.0	100.0

Concerning volume, glass packaging varies considerably in size. However, in weight, 46.0% of glass was 100 ml packaging, and 67.3% of all glass was represented by combining 100, 200, 250 and 500 ml packaging.

Regarding plastic packaging, polymer information was registered when feasible (i.e. the resin identification code and/or polymer name was visible on the packaging or label: 1 – PET (polyethylene terephthalate), 2 – HDPE, 3 – PVC (polyvinyl chloride), 4 – LDPE (low-density polyethylene), 5 – PP (polypropylene), 6 – PS (polystyrene) and 7 – Other). It was not possible to assure which polymer many packages were made of (43.8% of plastic VM packaging) since the packaging did not contain such information. Of those that could be identified, the HDPE stands out (32.5%, in weight), followed by PP, PET, LDPE and PS (8.9%, 8.1%, 4.0% and 0.2%, respectively, in weight). Other polymers represent 2.6%.

In particular, around 84.7% of total HDPE packaging labels indicated units of volume (registered or converted in ml units), and 14.3% related to weight (registered or converted in g units). Analysing volume sizes, HDPE packaging varies considerably. Packaging with 5,000 ml represents 56.4% of all HDPE observed, rising to 84.9% when also including volumes of 250 and 1,000 ml.

#### Assessment of the anatomical therapeutic and chemical groups for medicines

In the characterisation campaign, VM commercial names were registered to classify them into ATCVet medicines groups and analyse distribution by group (Table 4). All ATCVet medicines groups are represented, except for various ATCVet with the code QV (various). The results demonstrate that anti-infectives for systemic use (QJ) are predominant (34.0%, in weight, and 36.9%, in number). The other two groups that stand out are immunologicals (QI) (18.3%, in weight, and 28.6%, in number), and alimentary tract and metabolism medicines (QA) (12.7%, in weight, and 10.9%, in number).

**Table 4.** Veterinary medicines packaging waste, by veterinary medicines, in the Valormed subsystem.

Categories	Quantity (%)	
	In weight	In number
Anti-infectives for systemic use (QJ)	34.0	36.9
Immunologicals (QI)	18.3	28.6
Alimentary tract and metabolism (QA)	12.7	10.9
Genito-urinary system and sex hormones (QG)	4.0	6.8
Blood and blood forming organs (QB)	8.1	3.7
Systemic hormonal preparations^a^ (QH)	2.7	3.2
Musculo-skeletal system (QM)	6.4	2.3
Nervous system (QN)	1.5	0.9
Antiparasitic products, insecticides and repellents (QP)	2.0	0.6
Cardiovascular system (QC)	0.6	0.4
Dermatologicals (QD)	0.5	0.3
Respiratory system (QR)	0.5	0.2
Sensory organs (QS)	<0.1	<0.1
Antineoplastic and immunomodulating agents (QL)	<0.1	<0.1
Veterinary biocidal products (VBP)	0.1	<0.1
No data	8.5	5.2
Total	100.0	100.0

a Excluding sex hormones and insulins.

No data group is associated with three different cases: VM packaging that did not present any label for identification, or was damaged, not allowing for its identification; VM packaging waste that appeared in pieces, not allowing for its identification and VM commercial names which did not appear on the ATCVet database because they are not considered as VM, for example food supplements.

#### Assessment of the source segregation in biological and pharmacological containers

To examine waste segregation by producers, the distribution of VM groups in each type of collection container was analysed (Table 5), with reference to the Valormed requirements. All immunological VM packaging waste should be put in biological containers by producers, as these VM are considered biohazardous waste. Other VM packaging waste should be put in pharmacological containers.

**Table 5.** Veterinary medicines packaging waste by veterinary medicines in each type of collection container.

Categories		Quantity (%)	
		In weight	In number
Biological containers	Immunologicals (QI)	98.8	99.7
	Anti-infectives for systemic use (QJ)	1.0	0.3
	Musculo-skeletal system (QM)	0.2	<0.1
	Total	100.0	100.0
Pharmacological containers	Anti-infectives for systemic use (QJ)	35.8	41.0
	Immunologicals (QI)	13.9	20.6
	Alimentary tract and metabolism (QA)	13.4	12.1
	Genito-urinary system and sex hormones (QG)	4.3	7.6
	Blood and blood forming organs (QB)	8.6	4.1
	Systemic hormonal preparations^a^ (QH)	2.9	3.5
	Musculo-skeletal system (QM)	6.7	2.5
	Nervous system (QN)	1.6	1.0
	Antiparasitic products, insecticides and repellents (QP)	2.1	0.6
	Cardiovascular system (QC)	0.7	0.5
	Dermatologicals (QD)	0.5	0.3
	Respiratory system (QR)	0.5	0.2
	Sensory organs (QS)	<0.1	<0.1
	Antineoplastic and immunomodulating agents (QL)	<0.1	<0.1
	Veterinary biocidal products (VBP)	0.1	<0.1
	No data	8.9	5.8
	Total	100.0	100.0

a Excluding sex hormones and insulins.

In the characterisation campaign, it was found that immunological VM packaging waste represented almost the whole content of biological containers, whether measured in weight (98.8%) or in number (99.7%). This means that the livestock producers who separate this type of packaging waste are doing it correctly (a relevant subject, as substantiated by Koytcheva et al. (2021) and Rees et al. (2021)).

Regarding pharmacological containers, VM packaging waste from anti-infectives for systemic use is one of the most relevant and represents 35.8%, measured in weight, and 41.0% measured in number. However, the second most predominant VM group is immunologicals and points to livestock producers incorrectly following procedures, due to lack of knowledge of source segregation and/or negligence. This group represents 13.9%, measured in weight, and 20.6%, measured in number, indicating a need to increase the awareness of these producers (findings supported by the results of Kramer et al. (2020) and Qasmi and Khan (2019)). Alimentary tract and metabolism VM is the third most important group in pharmacological containers. Quantities related to this group are 13.4%, measured in weight, and 12.1%, measured in the number of units.

### Potential for reduction and recycling

#### Potential for reduction

During this research, based on frequent contact with the PRO responsible for this waste subsystem, representing the producers, and the characterisation campaigns executed, the potential for a reduction in VM packaging appears to be very low or not viable. This is partly not only due to design and safety issues, but also because a small amount of VM remnants were detected during the characterisation campaign (1.1% of the total sample, measured in weight). Initially, the intention was to understand the design of VM packaging, forming conclusions regarding the need to resize individual packaging or packaging units, change material type or other relevant aspects. However, regulations concerning changes in VM packaging are complex and persistent (as demonstrated by Schmerold et al. (2023)), due to safety issues around protecting active substances. Furthermore, this type of change involves investment in research and the alteration of most packaging established in the market. However, more research initiatives are needed to tackle the increasing problem of packaging waste, independently of its source, in order to realise a circular economy (European Commission, 2020).

By examining VM remnants, to study the need to adjust packaging sizes to consumer’s needs and reduce VM packaging waste, they were found to represent a very low quantity of the total sample, so it is not feasible to reduce waste through this criterion.

#### Potential for recycling

Glass is the most representative material in VM packaging collected by Valormed in Portugal and its recycling rate is currently very low. This might be because most packaging has a metal band and a rubber seal, which may cause difficulties with current recycling processes, although the glass recyclers contacted during this research stated that recycling packaging with these characteristics is achievable. Another reason could be that 21.5% of glass packaging waste in this waste subsystem has a volumetry lower than 100 ml, making the sorting process difficult.

The characterisation campaign revealed that immunological VM packaging waste in pharmacological containers accounted for 13.9%, measured in weight, of the total sample. There are doubts about the need to have a different container (biological) to solely collect immunological VM packaging waste. Regarding the current legal framework in Portugal, it is mandatory to either incinerate this type of waste or to subject it to a pre-treatment process (e.g. autoclaving), and then dispose of it in a landfill for non-hazardous waste.

This option is very specific to the Portuguese legal framework, which classifies this waste as a biohazardous risk, requiring specific treatment, a recommendation made by the national authority on waste and DGAV, based on a precautionary principle, as risk tests were never carried out. Furthermore, as some of this packaging waste appears in the pharmacologic containers, and if the biohazard risk is real, it will be necessary to promote awareness campaigns for stakeholders (as stated by Koytcheva et al. (2021), kramer et al. (2020) and Qasmi and Khan (2019)), to ensure better source segregation.

Although the literature on this subject is scarce, Martinho and Gonçalves (2000) pointed out that in Germany and the United States, for instance, the glass packaging from antibiotics, immunological or other medicines is considered a material of very good quality and is accepted without any restrictions.

The current existing barriers, the characteristics of the sorting centres and the Portuguese national legal framework make it more difficult for Valormed to reach the obligatory recycling target, although it could potentially reach 54.3%, based on the VM packaging waste characterisation campaign executed here, and also the mass balance. These recommendations are being analysed by the respective PRO to promote awareness campaigns for stakeholders, reflecting current system logistics, and, if feasible, increasing recycling rates to achieve the targets imposed by national authorities, but without compromising on safety and environmental conditions.

## Conclusions

The existing scientific literature on VM packaging waste is rather limited and lacks, to the best of the authors’ knowledge, results about physical characterisation campaigns regarding VM packaging waste, particularly within the European context. Such data are crucial for supporting the diagnosis and the subsequent decision-making processes regarding the implementation of EPR schemes dedicated to VM packaging waste. It is also especially important for evaluating the principles of the circular economy in relation to waste management, using robust and comparable indicators across different countries or regions. To address this knowledge gap, a protocol was created for this specific waste stream, considering the waste entering the sorting centre, but also the refuse.

According to the results of the mass balance, there is a significant loss of material, it being sent for disposal, that could otherwise be recycled if legal requirements and other practices were reconsidered. In this case, it is necessary to better understand, through future research, what the real biohazard risk of VM packaging waste is to encourage a serious reflection, involving national authorities and the stakeholders involved in the VM packaging waste circuit, about the extent of the restrictions on the recycling of VM waste. In general, this is particularly relevant in the case of glass, which is the predominant material encountered in the VM waste characterised.

The potential for a reduction in waste generated is considered very low or not viable mainly due to design and safety issues, which have to comply with strict regulations. Recycling could be improved, according to the results obtained, as immunological medicines are frequently collected in pharmacological containers, although they ought to be collected in biological containers. Therefore, reinforcing training and awareness campaigns regarding source segregation and how to handle biohazardous waste could improve the situation. In the meantime, biohazards should be studied in detail to prove whether immunological VM are a tangible risk and subsequently viable or not for recycling processes.

The existing ambitious recycling targets defined by Portuguese authorities for this waste stream have to be considered in terms of understanding the various regulatory constraints that VM faces and the existing constraints in sorting centres. It may be worth assessing the possibility of lowering recycling targets for this subsystem. Regardless of such changes, communication and training initiatives must be encouraged and implemented so that this vision is integrated and involves all stakeholders, improving all the processes and contributions. The results obtained prove that glass, being the predominant packaging material, ought to be considered initially because it could allow for a significant increase in the recycling potential of VM waste. Furthermore, recycling the glass from VM packaging waste is a viable process, considering the results of the authors’ consultation with the Portuguese glass recycling industry.

Given these results, there are two specific recommendations for future research on this theme: firstly, to carry out a biological risk analysis of immunological VM glass packaging waste, and secondly to carry out an operational assessment of the recycling process of glass from VM packaging waste that has rubber and metal attached. This is relevant when considering the Regulation (EU) 2019/6, of 11 December 2018, on veterinary medicinal products, establishing that the European Member States shall provide adequate systems to collect and dispose of the waste resulting from this activity.
